# Lipopolysaccharide-Deficient *Acinetobacter baumannii* Due to Colistin Resistance Is Killed by Neutrophil-Produced Lysozyme

**DOI:** 10.3389/fmicb.2020.00573

**Published:** 2020-04-17

**Authors:** Go Kamoshida, Takuya Akaji, Norihiko Takemoto, Yusuke Suzuki, Yoshinori Sato, Daichi Kai, Taishi Hibino, Daiki Yamaguchi, Takane Kikuchi-Ueda, Satoshi Nishida, Yuka Unno, Shigeru Tansho-Nagakawa, Tsuneyuki Ubagai, Tohru Miyoshi-Akiyama, Masataka Oda, Yasuo Ono

**Affiliations:** ^1^Department of Microbiology and Infection Control Sciences, Kyoto Pharmaceutical University, Kyoto, Japan; ^2^Department of Microbiology and Immunology, School of Medicine, Teikyo University, Tokyo, Japan; ^3^Pathogenic Microbe Laboratory, Department of Infectious Diseases, Research Institute, National Center for Global Health and Medicine, Tokyo, Japan

**Keywords:** *Acinetobacter baumannii*, colistin, LPS, lysozyme, neutrophil

## Abstract

*Acinetobacter baumannii* causes nosocomial infections due to its multidrug resistance and high environmental adaptability. Colistin is a polypeptide antibacterial agent that targets lipopolysaccharide (LPS) and is currently used to control serious multidrug-resistant Gram-negative bacterial infections, including those caused by *A. baumannii*. However, *A. baumannii* may acquire colistin resistance by losing their LPS. In mouse models, LPS-deficient *A. baumannii* have attenuated virulence. Nevertheless, the mechanism through which the pathogen is cleared by host immune cells is unknown. Here, we established colistin-resistant *A. baumannii* strains and analyzed possible mechanisms through which they are cleared by neutrophils. Colistin-resistant, LPS-deficient strains harbor mutations or insertion sequence (IS) in *lpx* genes, and introduction of intact *lpx* genes restored LPS deficiency. Analysis of interactions between these strains and neutrophils revealed that compared with wild type, LPS-deficient *A. baumannii* only weakly stimulated neutrophils, with consequent reduced levels of reactive oxygen species (ROS) and inflammatory cytokine production. Nonetheless, neutrophils preferentially killed LPS-deficient *A. baumannii* compared to wild-type strains. Moreover, LPS-deficient *A. baumannii* strains presented with increased sensitivities to antibacterial lysozyme and lactoferrin. We revealed that neutrophil-secreted lysozyme was the antimicrobial factor during clearance of LPS-deficient *A. baumannii* strains. These findings may inform the development of targeted therapeutics aimed to treat multidrug-resistant infections in immunocompromised patients who are unable to mount an appropriate cell-mediated immune response.

## Introduction

*Acinetobacter baumannii* is an aerobic Gram-negative bacillus that is widely distributed in nature. *A. baumannii* tolerates both damp and dry environments and proliferates easily in hospitals and on medical devices, causing nosocomial infections (Dijkshoorn et al., 2007; Maragakis and Perl, 2008; Munoz-Price and Weinstein, 2008). On the healthy human skin, *A. baumannii* is usually harmless; however, it may cause opportunistic infections in immunocompromised patients (Chu et al., 1999; Dijkshoorn et al., 2007; Maragakis and Perl, 2008; Munoz-Price and Weinstein, 2008). *A. baumannii* infection is characterized by pneumonia, sepsis, urinary tract infection, meningitis, and other conditions. The bacteria have been implicated as the source of ventilator-associated pneumonia (VAP) and urinary tract and blood vessel infections from contaminated catheters and surgical wound infections (Cisneros and Rodríguez-Baño, 2002; Garnacho-Montero et al., 2005; Dijkshoorn et al., 2007; Maragakis and Perl, 2008; Munoz-Price and Weinstein, 2008). *A. baumannii* is sometimes responsible for outbreaks among ICU (intensive care unit) patients. This pathogen requires constant surveillance in health care institutions (Garnacho-Montero et al., 2015; Leao et al., 2016).

The pathogenicity of *A. baumannii* may be explained by its bacterial capsule, biofilm formation, secretory systems, high adhesion capacity, strong affinity for iron, and other factors not yet identified (Mcconnell et al., 2013; Weber et al., 2016; Garcia-Patino et al., 2017; Wong et al., 2017). Previously, we investigated the interaction of *A. baumannii* with neutrophils and found that it inhibits neutrophil extracellular traps (NETs), a neutrophilic biological defense mechanism that traps and inactivates bacteria (Kamoshida et al., 2018). Moreover, we found that *A. baumannii* spreads throughout the body by flagging and hijacking neutrophils in a process known as “bacterial immunity taxi” (Kamoshida et al., 2015, 2016). Thus, it is necessary to study the interaction between *A. baumannii* and host immune cells for a better understanding of this bacterial pathogen.

*Acinetobacter baumannii* rapidly and readily acquires drug resistance. It harbors a gene encoding β-lactamase, has poor outer membrane permeability to antibacterial agents, acquires resistance through foreign genes harbored by plasmids, and readily undergoes biofilm formation (Perez et al., 2007; Peleg et al., 2008). Nosocomial infections caused by *A. baumannii* have become severe due to the high rate of drug resistance in the organism. Multidrug-resistant *A. baumannii* (MDRA) has proliferated and dispersed globally. Despite the close attention of medical professionals, MDRA is still difficult to manage and remains a critical issue. The number of fatalities ascribed to *A. baumannii* infections continues to rise worldwide (Dijkshoorn et al., 2007; Peleg et al., 2008).

Colistin is a polypeptide antibacterial drug that targets lipopolysaccharide (LPS) on the surfaces of Gram-negative bacteria and is the last resort against serious infections caused by multidrug-resistant Gram-negative bacteria such as MDRA (Levin et al., 1999; Falagas and Kasiakou, 2005; Maragakis and Perl, 2008). *A. baumannii* acquires colistin resistance at a rate of 10^–8^–10^–9^ (Moffatt et al., 2010; Cai et al., 2012). In the event that colistin proves ineffective against MDRA, there may be no other drugs available to treat the infection. Colistin cannot be used efficaciously against MDRA without first elucidating its resistance mechanism. *A. baumannii* acquires resistance to colistin by completely losing its LPS (Moffatt et al., 2010). LPS deficiency in *A. baumannii* causes various changes in the organism, including its relative sensitivity to antibacterial drugs (Moffatt et al., 2010; Carretero-Ledesma et al., 2018). Compared to wild type, the virulence of LPS-deficient *A. baumannii* is low, as found in mouse models (Moffatt et al., 2013; Beceiro et al., 2014; Carretero-Ledesma et al., 2018). However, if and how immune cells contribute to bacterial clearance remains ill-defined.

Neutrophils are immune cells that play a pivotal role during the initial immune response to various bacterial infections. Neutrophils migrate toward infection sites and act against bacteria through phagocytosis and by producing toxic factors such as reactive oxygen species (ROS) (Nathan, 2006; Nauseef and Borregaard, 2014). Moreover, neutrophil granules have molecules, such as lysozyme, lactoferrin, LL-37, and elastase with antimicrobial activity that are important for immune defense against bacterial infections (Ellison and Giehl, 1991; Borregaard et al., 2007). Thus, understanding neutrophil–bacterial interaction is very important in controlling infection. In the present study, we established colistin-resistant strains to investigate effects of neutrophil-secreted lysozyme on LPS-deficient *A. baumannii* and examined clearance mechanism of this bacteria by neutrophils. Colistin-resistant *A. baumannii* (CRAb) are often isolated in clinical settings, particularly in ICU patients with immunodeficiency conditions such as neutropenia (Qureshi et al., 2015). This study may help to treat multidrug-resistant infections concluding colistin-resistant bacteria in immunocompromised patients who are unable to mount an appropriate cell-mediated immune response such as neutropenia.

## Materials and Methods

### Bacterial Strains, Plasmids, and Neutrophils

Wild-type *A. baumannii* ATCC 19606 was used as the reference strain (Kamoshida et al., 2015, 2016, 2018). Bacteria were grown in LB (Luria-Bertani) broth (BD Biosciences, San Diego, CA, United States) for 16 h at 37°C shaking. Colistin-resistant variants of ATCC 19606 (CRAb) were isolated by directly plating the parent strain onto LB agar containing 10 μg/ml colistin sulfate (Sigma-Aldrich Corp., St. Louis, MO, United States).

The pMU125 plasmid (*Escherichia coli*–*Acinetobacter* shuttle vector pWH1266 with *gfp*) was kindly provided by Dr. Luis Actis of Miami University, Miami, FL, United States (Dorsey et al., 2002). To construct the plasmid, pKAMO was prepared by In-Fusion cloning (Takara Bio Inc., Shiga, Japan), ampicillin resistance gene in pMU125 was replaced with kanamycin resistance gene from pHSG298 and *gfp* was replaced with a multiple cloning site sequence (5′-CTCGAGGAGCTCAGTACTGCGGCCGCAGGTACCGCA TGC-3′: *Xho*I–*Sac*I–*Sca*I–*Not*I–*Kpn*I–*Sph*I). *lpxC* DNA fragment (primer pairs: 5′-ACAGAACAGAATTCTATGGTG AAACAGCGTACTCTCA-3′ and 5′-TCTGAGATGGTCGACT TATGTCACACTCACGTATGGA-3′) from ATCC 19606 was ligated to pKAMO to construct the lpxC expression plasmid pKAMO-lpxC. CRAb-5 was transformed by electroporation with pKAMO-lpxC and pKAMO (control vector) to construct CRAb-LPS and CRAb-mock, respectively. The bacteria were selected on LB agar containing 50 μg/ml kanamycin.

Neutrophils were isolated from the peripheral blood of healthy human volunteers (*n* = 10) as previously described (Kamoshida et al., 2017). The heparinized blood was mixed with 1% (w/v) dextran (MW 200,000) in saline to sediment erythrocytes. The supernatant was subjected to density gradient centrifugation in a Lymphosepar I (Wako Pure Chemical Industries Ltd., Osaka, Japan). The neutrophils were purified from the cell pellets under hypotonic conditions to lyse any remaining erythrocytes. Neutrophil purity was >95%. Samples from independent donors were used for each set of experiments. Written informed consent was obtained from all study participants in accordance with the Declaration of Helsinki. The study was approved by the Ethical Review Committee of the School of Medicine of Teikyo University, Tokyo, Japan.

### Neutrophil and Bacterial Co-cultures

The bacteria were cultured on LB broth containing 50 μg/ml kanamycin at 37°C for 16 h, rinsed with phosphate-buffered saline (PBS), and resuspended in fresh RPMI 1640 (Sigma-Aldrich). Bacteria [MOI (multiplicity of infection) 50, 5 × 10^7^ CFU/ml] and neutrophils (10^6^ cells/ml) were co-cultured in RPMI 1640 containing 2% human serum and maintained in standing still suspension at 37°C under a 5% CO_2_ atmosphere for the indicated amount of time. When required by experiments, 5 μg/ml cytochalasin B (simultaneous addition) was used to inhibit neutrophil phagocytosis in suspension co-cultures.

### PCR-Sanger Sequencing

Genome regions containing *lpx* genes were amplified by colony PCR using the following primers: *lpxA*: 5′-TGGTAA TGCAGAAGCGCGGTATCTACAA-3′ and 5′-ATCCTCTAGA GTCGACCAATATTCAAAGTCTGAAGAAGCA-3′; *lpxC*: 5′- TCAGCAACGTAAGTAATTTAGCGTACA-3′ and 5′-GCCA AGCTTTACTACGTTTGGCAAGCAA-3′; *lpxD*: 5′-AAGCTT GCATGCGTTAAGCAAGCTGCTGAGCAATTACGAA-3′ and 5′-CCAATAAGAATGGGTAACGATGCGGCAA-3′.

The PCR products were treated with EXOSAP-IT (Thermo Fisher Scientific, Waltham, MA, United States) and followed by cycle-sequencing reaction with Big Dye Terminator v3.1 (Applied Biosystems, Foster City, CA, United States) using the primers listed above. Direct sequencing process was performed in 3730xl Genetic Analyzer (Applied Biosystems).

### *Limulus* Amebocyte Lysate Assay

Limulus amebocyte lysate assay was performed using a Toxicolor LS-50M set (Seikagaku Corporation, Tokyo, Japan), according to the manufacturer’s instructions. Overnight *A. baumannii* cultures (after adjusted OD_600_ = 0.1) were diluted in sterile, pyrogen-free water (Otsuka Pharmaceutical Co., Ltd., Tokyo, Japan).

### Growth Curve

Bacterial culture density was adjusted to OD_600_ = 0.005 from overnight cultures. The bacteria were grown in LB broth for 8 h at 37°C, shaking 135 rpm using BioShaker BR-23FP (Taitec, Saitama, Japan). Culture OD_600_ was measured every hour using BioPhotometer plus (Eppendorf, Hamburg, Germany).

### Lipopolysaccharide Analysis by Polyacrylamide Gel Electrophoresis (PAGE)

Lipopolysaccharide was purified from 16-h cultures (adjusted OD_600_ = 0.1) of *A. baumannii* using LPS Extraction kit (iNtRON Biotechnology, Seoul, South Korea), according to the manufacturer’s instructions. LPS preparations were separated on 15% (w/v) SDS-PAGE as previously described (Kamoshida et al., 2012). To visualize LPS, the gel was stained with 2D-Silver Stain Reagent II (Cosmo Bio, Tokyo, Japan).

### Determination of Minimum Inhibitory Concentrations

Minimum inhibitory concentrations for antimicrobial drugs and egg white lysozyme (Sigma-Aldrich), human lactoferrin (Sigma-Aldrich), LL-37 (Peptide Institute, Osaka, Japan), human serum, and H_2_O_2_ were determined by microdilution in LB broth according to the Clinical and Laboratory Standards Institute protocol (M07 Methods for Dilution Antimicrobial Susceptibility Tests for Bacteria That Grow Aerobically, 11th Edition). Bacterial culture density was adjusted to OD_600_ = 0.001 from overnight cultures and cultured for 16 h at 37°C. A twofold dilution series was used for each molecule to set up the MIC studies. The concentration range was between 256 and 0.007 μg/ml for antimicrobial drugs, 1000 and 0.002 μg/ml for lysozyme, 4000 and 4 μg/ml for lactoferrin, 5000 and 5 nM for LL-37, 40 and 0.02% for serum, and 10 and 0.01% for H_2_O_2_. Survival rates were calculated as percentages of the uninhibited pure bacterial culture.

### Detection of ROS and Superoxide

ROS and superoxide production levels in neutrophil and bacterial co-cultures were measured by flow cytometry (FACSCanto II, BD Biosciences) and a total ROS/superoxide detection kit (Enzo Life Sciences, Farmingdale, NY, United States). Neutrophils induced with 100 nM phorbol-12-myristate-13-acetate (PMA) served as positive controls.

### Measurements of Cytokine Levels

Cytokines were evaluated by BD cytometric bead array (CBA) system (BD Biosciences) [human inflammation kit with interleukin (IL)-8, IL-1β, IL-6, IL-10, tumor necrosis factor (TNF), and IL-12p70)]. Supernatants were obtained from suspension co-culture of neutrophils and bacteria. The supernatants of neutrophils stimulated with PMA (100 nM) served as positive controls.

### Lysozyme Measurement in Neutrophils

Lysozyme secreted by neutrophils into supernatants was measured by enzyme-linked immunosorbent assay (ELISA) (Assaypro, St. Charles, MO, United States). Supernatants were obtained from neutrophils co-cultured with bacteria (MOI 50) in RPMI 1640 containing 2% human serum at 37°C for 4 h in 5% CO_2_. Lysozyme was removed by mixing 1.0 ml of the neutrophil supernatant with 5 μg/ml anti-lysozyme antibody (Abcam, Cambridge, United Kingdom) conjugated with protein G–Sepharose [0.05 ml; 50% (w/v) suspension] and incubating at 4°C for 16 h. The supernatant was recovered by low-speed centrifugation.

### Bacterial Clearance by Neutrophils

Neutrophil and bacteria (MOI 50) were co-cultured for 4 h. Appropriate dilutions of co-culture suspension were spread onto LB agar plates and incubated overnight at 37°C to count bacteria colonies and evaluate cell viability. Bacterial clearance capacity of co-culture supernatants of neutrophils and CRAb-5 was assessed by using CRAb-LPS or CRAb-mock strains. Bacteria (5 × 10^7^ CFU/ml) were incubated for 4 h. Cell suspension was then inoculated onto LB agar plates containing kanamycin to count viable cells. To test bacterial clearance in supernatants without lysozymes produced by neutrophils, co-culture supernatants were cleared of lysozyme as described above, prior to use in clearance capacity experiments.

### Statistical Analysis

Data are expressed as the mean ± standard deviation (SD) and were compared by unpaired Student’s *t*-test and two-way ANOVA. Differences with *P* < 0.05 were considered statistically significant.

All methods were carried out in accordance with relevant guidelines and regulations.

## Results

### CRAb Strains Lack LPS and Exhibit Attenuated Growth

**FIGURE 1:**
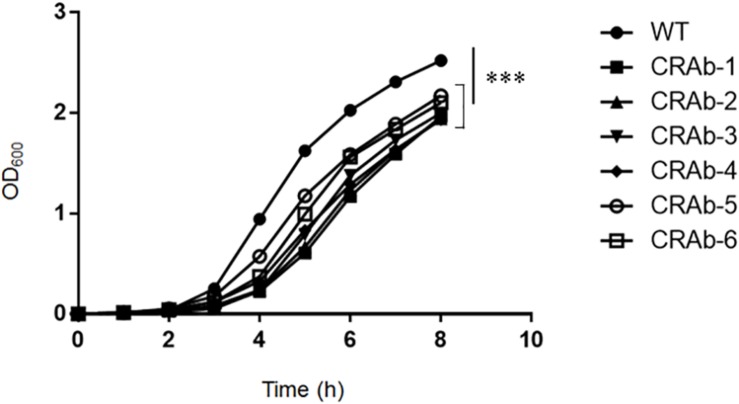
Growth curve of colistin-resistant *Acinetobacter baumannii* (CRAb) strains. ATCC 19606 (parent) and CRAb strains preadjusted to OD_600_ = 0.005 were grown in LB broth at 37°C for 8 h. OD_600_ was measured hourly. Data are means ± SD; *n* = 3 per group. ^∗∗∗^*P* < 0.001.

*Acinetobacter baumannii* acquires colistin resistance at a frequency in the order of 10^–8^–10^–9^. We used colistin to select for and establish six CRAb strains and analyzed *lpxA*, *lpxC*, and *lpxD* genes, previously reported to participate in colistin resistance (Moffatt et al., 2010). We confirmed the presence of non-synonymous mutation and/or gene rearrangement in some or all of *lpx* genes in all six CRAb strains (Table 1). CRAb-1, CRAb-2, and CRAb-5 presented with non-synonymous point mutations or depletions of intact genes. In CRAb-6, the IS*Aba11* sequence was inserted into *lpxC*, resulting in LpxC truncation at *C*-terminal amino acid 160 (Moffatt et al., 2011). In CRAb-3 and CRAb-4, *lpxC* or *lpxA* was not amplified, suggesting deletion of these regions or insertion of large insertion sequences. We measured bacterial endotoxin using the LAL assay. Endotoxin levels were below the detection limit in most of the CRAb strains (Table 1). Endotoxin production was detected only for CRAb-5 but at a considerably lower level than that of the wild type (ATCC 19606). Therefore, disruption of *lpxA*, *lpxC*, and *lpxD* genes in *A. baumannii* may have accounted for the observed LPS deficiency leading to colistin resistance. All CRAb strains grew slower than the wild type (Figure 1), indicating the importance of LPS synthetic pathway in normal growth.

**TABLE 1:** *Lpx* mutation analysis and limulus amebocyte lysate (LAL) assay of colistin-resistant *Acinetobacter baumannii* (CRAb) strains.

**Strain**	***IpxA***	***IpxC***	***IpxD***	**AA change**	**EU/ml**
ATCC19606	WT	WT	WT		69 × 10^3^ ± 27 × 10^3^
CRAb-1	382-384GTA→-	WT	WT	LpxA V128- (deletion of 128 valine)	0.2>
CRAb-2	WT	312T→A	934G→T	LpxD E312* (truncation of C-terminal 45 aa)_*T*_ LpxC D104E	0.2>
CRAb-3	WT	no band	WT	Not detected	0.2>
CRAb-4	No band	WT	WT	Not detected	0.2>
CRAb-5	WT	573T→G	WT	LpxC F191L	1.5 ± 1.2
CRAb-6	WT	420T::X	WT	LpxC D141* (truncation of C-terminal 160 aa)	0.2>

*AA: amino acid EU: endotoxin units.*

### Establishment of LPS Complementation Strain of CRAb

**FIGURE 2:**
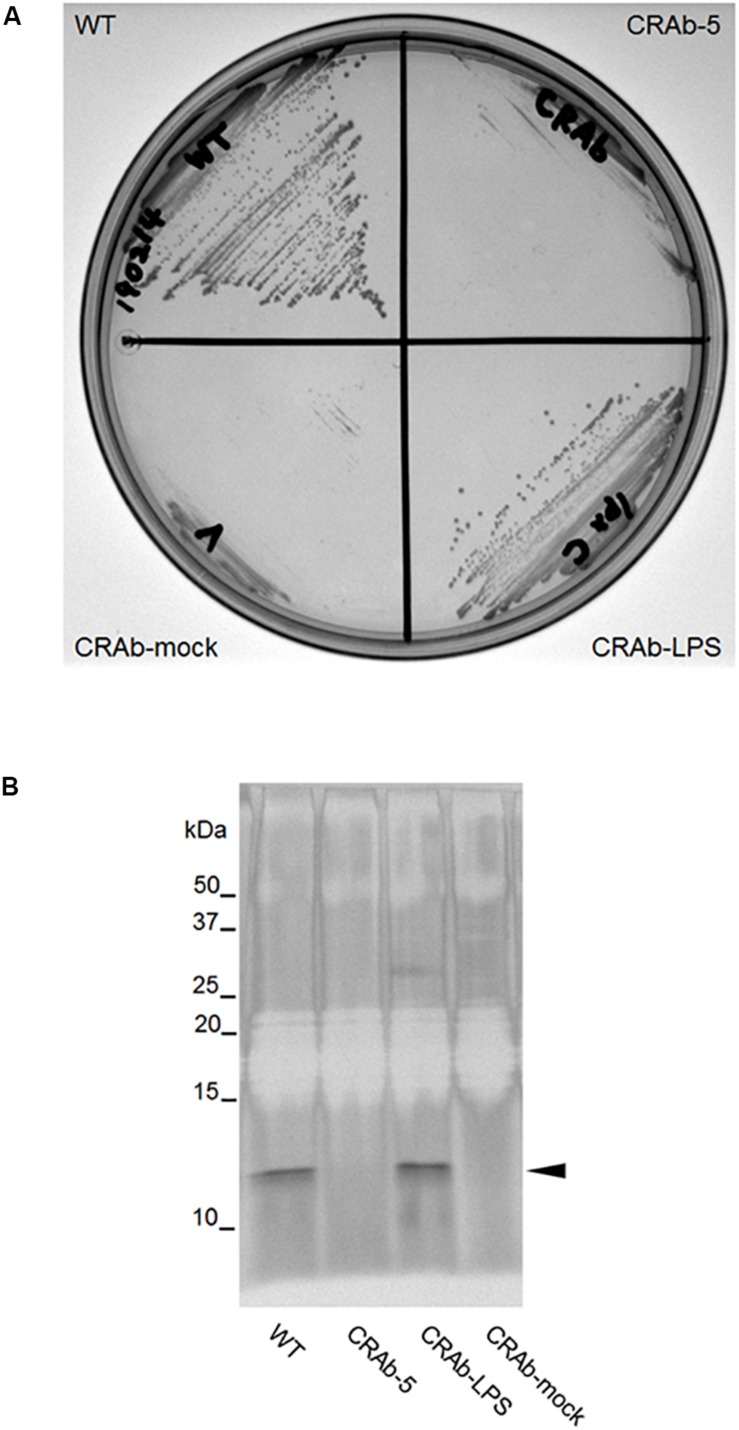
Confirmation of lipopolysaccharide (LPS) complementation strain of colistin-resistant *Acinetobacter baumannii* (CRAb). **(A)** LPS complementation strain colony formation. ATCC 19606 (WT), CRAb-5, CRAb-LPS, and CRAb-mock were streaked on LB agar plates, cultured at 37°C for 18 h to confirm colony formation. LPS was purified from colonies. LPS preparations were separated by electrophoresis and visualized by silver stain **(B)**. Arrow: lipid A.

It was predicted that CRAb-5 is deficient in intact *lpxC* and lost most of its LPS. To examine the differences between LPS-positive and LPS-negative *A. baumannii*, we constructed a complementation strain by re-introducing intact *lpxC* into CRAb-5. The complemented *lpxC* mutant (CRAb-LPS) recovered its displayed same colony size as the wild type (Figure 2A). The growth of CRAb-mock, a strain harboring the control vector, was similar to the parental CRAb-5 strain. To assess the capacity of the wild type, CRAb-5, CRAb-LPS, and CRAb-mock to produce LPS, we purified and analyzed LPS from their cultures. Wild-type and CRAb-LPS produced migrating lipid A in the molecular mass range of 10–15 kDa (Moffatt et al., 2010). CRAb-5 and CRAb-mock produced no detectable lipid A (Figure 2B). Thus, CRAb-LPS, the *lpxC* complementation strain, recovered LPS production and clarified the difference between LPS-positive and LPS-negative *A. baumannii*.

### MICs of Various Antimicrobial Drugs Against LPS-Deficient *A. baumannii* Strains

First, we evaluated the MICs of polymixins (colistin and polymyxin B). LPS-negative CRAb-5 and CRAb-mock were highly resistant to both drugs. In contrast, wild-type *A. baumannii* (ATCC 19606) and the LPS-restoring CRAb-LPS were sensitive to both drugs (Table 2). Moreover, susceptibility to β-lactams, such as meropenem, imipenem, and cefozopran, drugs that target peptidoglycan biosynthesis, was higher in CRAb-5 and CRAb-mock than the LPS-positive strains. The LPS-negative strains were also more sensitive to aminoglycosides such as amikacin. However, CRAb-5 and CRAb-mock were not more sensitive than their LPS-positive counterparts to tetracyclines such as minocycline and tigecycline, sulbactam, or the quinolone ciprofloxacin (Table 2). Thus, LPS deficiency in *A. baumannii* not only causes the organism to acquire colistin and polymyxin B resistance but also increases its sensitivity to β-lactams and several other antibiotics.

**TABLE 2:** Minimum inhibitory concentrations (MICs) of various antimicrobial drugs against lipopolysaccharide-deficient *Acinetobacter baumannii* strains.

	**WT**	**CRAb-5**	**CRAb-LPS**	**CRAb-mock**
	**ATCC19606**	**LPS-deficient**	**+intact IpxC**	**+control vector**
Colistin	0.5	>256	0.5	>256
Polymyxin B	0.25	128	0.5	64
Minocycline	0.25	0.125	0.25	0.06
Tigecycline	0.5	0.25	0.5	0.125
Sulbactam	0.5	0.5	0.5	0.5
Meropenem	0.5	0.007>	0.5	0.007>
Imipenem	8	1	8	1
Cefozopran	8	2	8	1
Amikacin	16	4	16	2
Ciprofloxacin	0.06	0.06	0.06	0.03

*Drug concentrations are in μg/ml.*

### LPS-Defective *A. baumannii* Stimulates Lower Levels of ROS and Inflammatory Cytokines in Neutrophils

**FIGURE 3:**
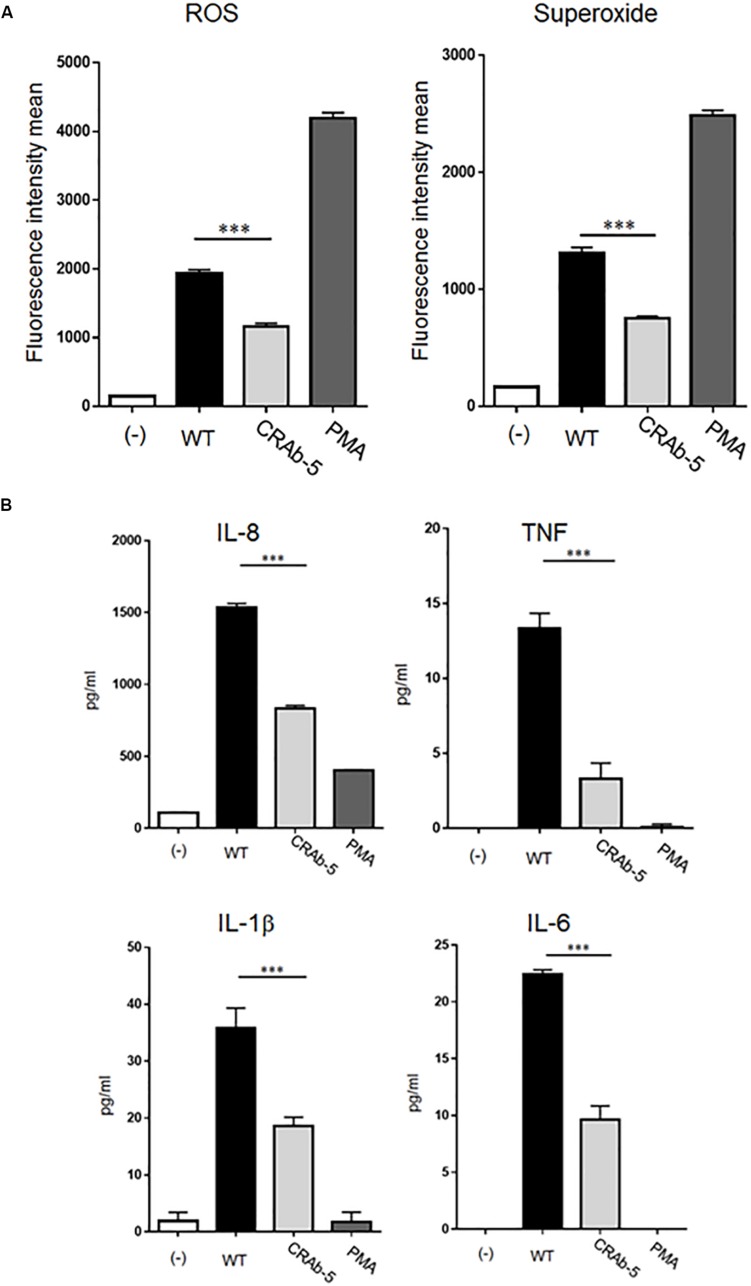
Measurements of inflammatory response molecules secreted by neutrophils. ROS and superoxide expression levels in neutrophils challenged by bacteria (ATCC 19606: WT, colistin-resistant *A. baumannii*: CRAb-5) were measured by flow cytometry and a total ROS/superoxide detection kit **(A)**. PMA (100 nM)-stimulated neutrophils served as positive controls. Data are means ± SD; *n* = 5 per group. ^∗∗∗^*P* < 0.001. **(B)** Inflammatory cytokines such as interleukin (IL)-8, tumor necrosis factor (TNF), IL-1β, and IL-6 produced by neutrophils in response to bacterial stimulation were measured by the cytometric bead array system. Supernatants were obtained by co-culturing neutrophils and bacteria for 4 h. The supernatants of neutrophils alone stimulated with PMA (100 nM) served as positive controls. Data are means ± SD; *n* = 4 per group. ^∗∗∗^*P* < 0.001.

Studies in mouse models demonstrated that LPS-deficient *A. baumannii* is less virulent than wild-type strains (Moffatt et al., 2013; Beceiro et al., 2014; Carretero-Ledesma et al., 2018). However, the mechanism of immune clearance of LPS-deficient bacterial pathogens like *A. baumannii* is not known. Based on the important roles of neutrophils in initial host immune responses, we examined interactions between neutrophils and LPS-deficient *A. baumannii*. First, we evaluated ROS and superoxide production in neutrophils exposed to *A. baumannii* and found lower levels in neutrophils induced by CRAb-5 than in those challenged by wild-type *A. baumannii* (Figure 3A). Similar results were obtained with CRAb-3 and CRAb-6 (Supplementary Figure S1). Furthermore, ROS levels were lower in neutrophils induced by CRAb-mock (empty vector control strain) than those by the LPS-restoring strain, CRAb- LPS (Supplementary Figure S2). These observations remained the same in experiments performed with formalin-killed bacteria (Supplementary Figure S3).

We next assessed inflammatory cytokines production by *A. baumannii*-stimulated neutrophils. Interleukin (IL)-8, tumor necrosis factor (TNF), IL-1β, and IL-6 levels were lower in neutrophils challenged with CRAb-5 than in those exposed to the wild type (Figure 3B). IL-10 and IL-12p70 were below detection limits. Collectively, these results suggest that LPS- deficient CRAb-5 strain is less effective than the parent strain at stimulating neutrophilic ROS, superoxide, and inflammatory cytokine production.

### LPS-Deficient *A. baumannii* Strains Are More Prone to Neutrophil Clearance

**FIGURE 4:**
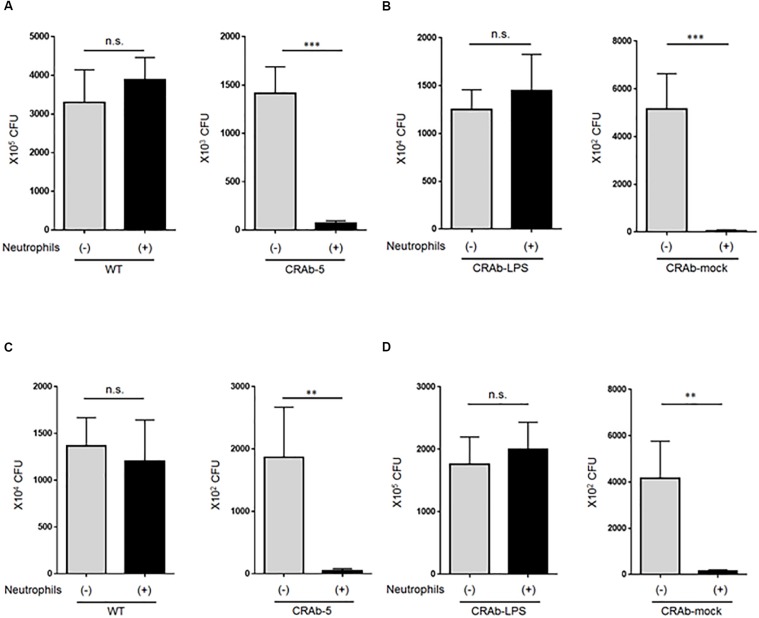
Assay of lipopolysaccharide-deficient *Acinetobacter baumannii* strain clearance by neutrophils. **(A)** ATCC 19606 (WT), colistin-resistant *A. baumannii* (CRAb-5), **(B)** CRAb-LPS, or CRAb-mock and neutrophils were co-cultured for 4 h. Surviving bacteria were then counted. In panel **(C)**, cytochalasin B was used to inhibit neutrophil phagocytosis in co-cultures. Clearance capacity of 4-h neutrophil/CRAb-5 co-culture supernatant was evaluated by adding supernatant to CRAb-LPS or CRAb-mock and incubating for 4 h. Viable cells were then counted by plating on kanamycin agar **(D)**. Data are means ± SD; *n* = 6 per group. n.s., not significant, ^∗∗∗^*P* < 0.001, ^∗∗^*P* < 0.01. Means are representative of three experiments.

To explore the impact of neutrophils on *A. baumannii* clearance, we first asked whether LPS-deficient CRAb-5 could still be killed by neutrophils. Wild-type *A. baumannii* or CRAb-5 and neutrophils were co-cultured for 4 h and the surviving bacteria were counted. We found that CRAb-5 was significantly killed by neutrophils, whereas the wild type was not (Figure 4A). CRAb-3 and CRAb-6 strains were also significantly killed by neutrophils (Supplementary Figure S4). Notably, CRAb-mock were killed by neutrophils but not CRAb-LPS (Figure 4B). We then repeated bacterial clearance experiments in the presence of neutrophil phagocytosis inhibition or with using neutrophil-CRAb-5 co-cultivation supernatants. The suppression of neutrophil phagocytosis by cytochalasin B did not prevent killing of CRAb-5 killing (Figure 4C). The observation of neutrophil-CRAb-5 co-cultivation resulted in nearly no phagocytosis of the pathogen (Supplementary Figure S5). Further, bacterial culture with co-culture supernatant killed LPS-deficient CRAb-mock but not LPS-restoring CRAb-LPS (Figure 4D). These results suggest that neutrophils killed LPS-deficient *A. baumannii* strains but not the wild type, with released factors present in neutrophils supernatant.

### LPS-Deficient *A. baumannii* Strains Differentially Respond to Various Neutrophilic Bactericidal Molecules

**FIGURE 5:**
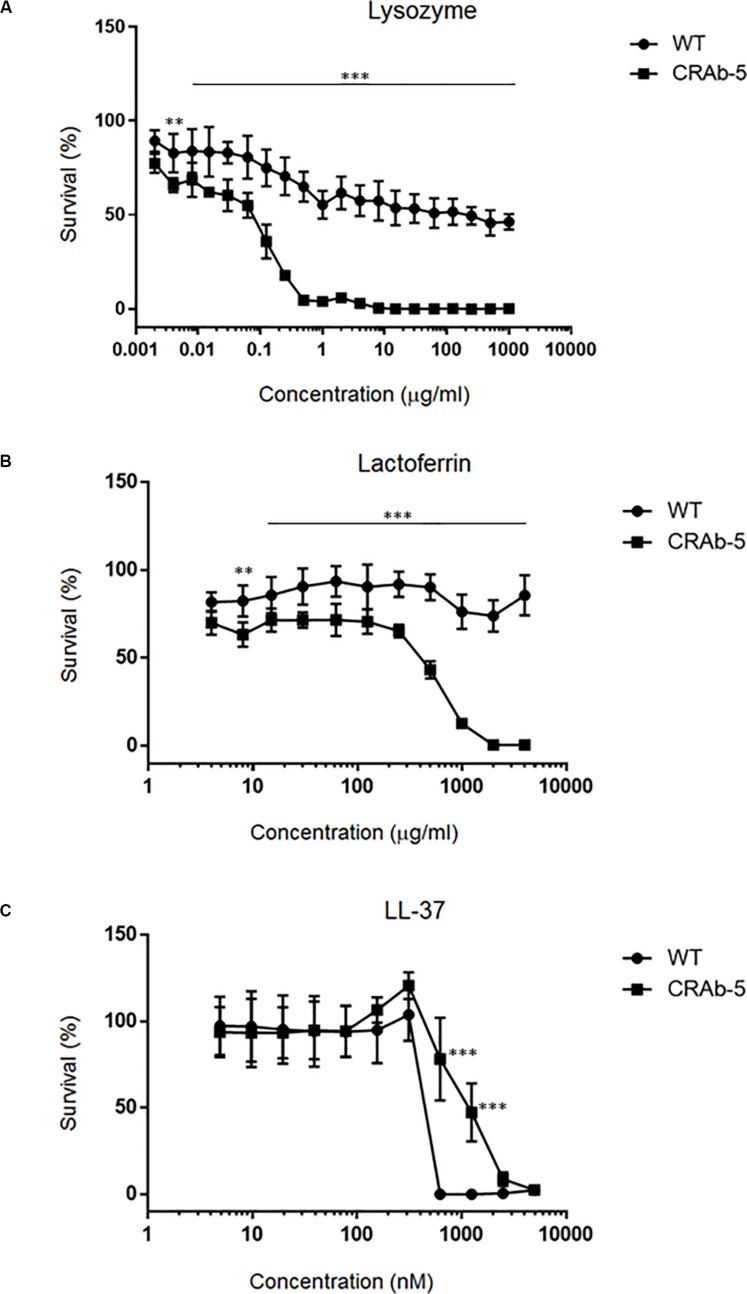
Dose–response curves of bactericidal ability of various biomolecules against lipopolysaccharide-deficient *Acinetobacter baumannii* strains. A twofold dilution series of **(A)** lysozyme (maximum concentration 1000 μg/ml), **(B)** lactoferrin (maximum concentration 4000 μg/ml), or **(C)** LL-37 (maximum concentration 5000 nM) was prepared and the viabilities of the ATCC 19606 (wild type) or colistin-resistant *A. baumannii* strain (CRAb-5) were assessed. Survival rates were calculated as percentages of the uninhibited, pure bacterial culture. Data are means ± SD; *n* = 3 per group. ^∗∗∗^*P* < 0.001, ^∗∗^*P* < 0.01.

Since neutrophils evidently secreted extracellular substances lethal to LPS-negative *A. baumannii* strains (Figure 4D), we examined bactericidal effects of known neutrophilic factors on LPS-deficient *A. baumannii*. The bactericidal activities of lysozyme and lactoferrin were higher against LPS-deficient *A. baumannii* (CRAb-5, CRAb-mock) than they were against LPS-positive wild type and CRAb-LPS. LPS-deficient CRAb-5 was killed by only 0.6 μg/ml lysozyme, whereas the wild type survived 1000 μg/ml lysozyme (Figure 5A). Lactoferrin killed CRAb-5 at 1000 μg/ml while the wild type survived 4000 μg/ml (Figure 5B). In contrast, the antibacterial activity of the cathelicidin peptide LL-37 was weaker against LPS-deficient than it was against LPS-positive *A. baumannii* strains. LL-37 killed the wild type at 625 nM but CRAb-5 survived 2500 nM LL-37 (Figure 5C). The resistance to serum was slightly lower in the LPS-negative *A. baumannii* strains. The bactericidal efficacies of H_2_O_2_ were nearly the same against both LPS-positive and LPS-negative *A. baumannii* (Table 3).

**TABLE 3:** Bactericidal activity of various biomolecules against lipopolysaccharide-deficient *Acinetobacter baumannii* strains.

	**WT**	**CRAb-5**	**CRAb-LPS**	**CRAb-mock**
Lysozyme	>1000 μg/ml	0.6 μg/ml	>1000 μg/ml	0.6 μg/ml
Lactoferrin	>1000 μg/ml	1000 μg/ml	>1000 μg/ml	1000 μg/ml
LL-37	625 nM	2500 nM	625 nM	1250 nM
Human-Serum	40%	20%	20%	10%
H_2_0_2_	0.02%	0.02%	0.02%	0.02%

### Neutrophil-Secreted Lysozyme Kills LPS-Deficient *A. baumannii* Strains

**FIGURE 6:**
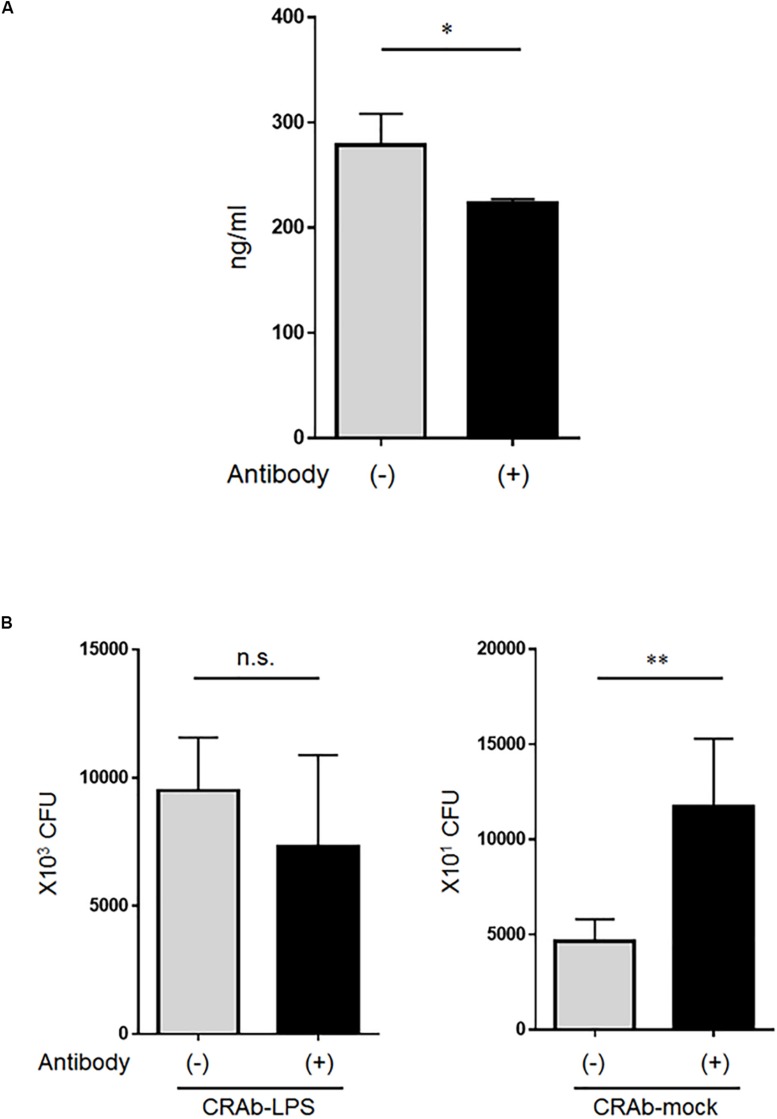
Effect of lysozyme released by neutrophils on lipopolysaccharide-deficient *Acinetobacter baumannii* strains. **(A)** Neutrophilic lysozyme secreted into culture supernatants was measured by ELISA. Supernatants were obtained from neutrophils co-cultured with colistin-resistant *A. baumannii* for 4 h. Lysozymes were neutralized in other supernatants with anti-lysozyme antibody. Data are means ± SD; *n* = 4 per group. **(B)** Bacterial clearance experiments were performed using these supernatants. The supernatants were added to colistin-resistant *A. baumannii* (CRAb)-LPS or CRAb-mock and viable bacteria were counted by plating on kanamycin agar. Data are means ± SD; *n* = 6 per group. n.s., not significant, ^∗∗^*P* < 0.01, ^∗^*P* < 0.05. Means are representative of three experiments.

Whereas low lysozyme concentrations killed LPS-deficient *A. baumannii* strains, even high lysozyme concentrations had no apparent adverse effect on the LPS-positive strains. Therefore, we speculated that the bactericidal factor in the neutrophil supernatant may have been lysozyme. Lysozyme at ∼280 ng/ml was detected in the co-cultured bacteria (CRAb-5)/neutrophil supernatant and reacting the supernatant with anti-lysozyme antibody reduced its amount (Figure 6A). Bacterial clearance experiments were conducted on these supernatants to determine the effects of lysozyme on LPS-deficient *A. baumannii*. Treatment of co-culture supernatant with anti-lysozyme decreased the killing effects on LPS-deficient CRAb-mock but had no apparent deleterious effects on LPS-restoring CRAb-LPS (Figure 6B). This observation suggests that neutrophilic lysozyme sufficiently kills LPS-deficient *A. baumannii*.

## Discussion

In the present study, we established CRAb strains and analyzed the mechanism through which neutrophils clear them. Previous studies reported that relative to the wild type, the virulence of LPS-deficient *A. baumannii* is low in mouse models (Moffatt et al., 2013; Beceiro et al., 2014; Carretero-Ledesma et al., 2018). This was based on the comparatively reduced inflammatory cytokine (TNF-α and IL-6) levels in the blood of mice subjected to the LPS-deficient *A. baumannii* (Carretero-Ledesma et al., 2018). Moreover, murine macrophage-like cell lines lacking receptors indicated that LPS-deficient *A. baumannii* received only weak NF-κB signals from TLR4 (Moffatt et al., 2013). It is known that neutrophils play an important role in the control of bacterial infection. However, the mechanism involved has not yet been elucidated. With a focus on the interaction between neutrophils and LPS-deficient *A. baumannii*, we found that neutrophils significantly killed LPS-deficient *A. baumannii* better than wild-type strains. Furthermore, LPS-deficient *A. baumannii* strains have increased susceptibility to antibacterial proteins and β-lactam antibacterial drugs and might be less able to survive *in vivo* due to reduced proliferation ability. Inflammatory cytokine production by neutrophils was relatively low in response to challenge induced by LPS-deficient strains, suggesting that these strains are lesser activators of neutrophils than wild-type *A. baumannii*. In LPS-deficient *A. baumannii* strains, it is conceivable that a decrease in the induction of inflammatory cytokines would minimize subsequent immune responses. This minor attenuation of immune response by LPS-deficient bacteria may not be a problem in healthy people; however, in patients with immune diseases such as neutropenia, infection may be worsened due to lesser neutrophil activation and reduced inflammatory cytokine production.

Lysozyme occurs in the cytoplasmic granules of macrophages and neutrophils (Leffell and Spitznagel, 1972). It catalyzes the hydrolysis of peptidoglycan, which is a major component of bacterial cell walls. Bacterial cell walls subjected to lysozyme lose their integrity and the cell undergoes lysis (Kirby, 2001). The LPS in the cell walls of Gram-negative bacteria inhibits lysozyme binding and prevents lysis (Ohno and Morrison, 1989; Brandenburg et al., 1998). Furthermore, LPS, the outer membrane of Gram-negative bacteria, serves as a selectively permeable barrier that keeps large molecules, such as lysozyme, from accessing periplasmic peptidoglycan (Nikaido, 2003). Loss of LPS likely leads, which in turn leads to increased access of lysozyme to periplasmic peptidoglycan. Wild-type *A. baumannii* and the complementary strains derived from it were less susceptible to lysozyme because of their LPS content. In contrast, CRAb-5 and CRAb-mock lacked LPS and were easier targets for lysozyme. Lysozyme has enhanced activity against colistin-resistant strains lacking LPS (Garcia-Quintanilla et al., 2014). Using complementary strains and neutrophil-derived lysozyme, we demonstrated that bacterial clearance by the enzyme was influenced by bacterial LPS status. Further, high concentrations of the antibacterial biomolecule lactoferrin killed only LPS-deficient *A. baumannii* strains. The primary function of lactoferrin is to sequester free iron, which is an essential substrate for bacterial growth (Masson et al., 1969). Its secondary role is to bind bacterial wall LPS. This reaction alters membrane permeability, especially at high lactoferrin concentrations (Farnaud and Evans, 2003; Drago-Serrano et al., 2012). LL-37 is a cationic antimicrobial present in macrophage and neutrophil lysosomes (Turner et al., 1998; Durr et al., 2006). Two conflicting reports on CRAb strains lacking LPS demonstrated increased or decreased resistance to LL-37 (Moffatt et al., 2013; Garcia-Quintanilla et al., 2014). We found that LPS-deficient *A. baumannii* strains were resistant to LL-37 (Table 3 and Figure 5C). LL-37 is positively charged. Whereas wild-type *A. baumannii* strains has anionic LPS, LPS-deficient strains lack this negatively charged molecule, making it difficult for cationic LL-37 to bind to them. Reportedly, LPS-deficient *A. baumannii* strains change the compositions of the outer membrane and increase the amount of molecules such as lipoproteins (Henry et al., 2012; Boll et al., 2016). Recently, secondary compensatory mutations for LPS deficiency have been reported in *A. baumannii* (Powers and Trent, 2018). The observed differential responses of *A. baumannii* strains in our study to biomolecules might be due to subtle differences in the outer membrane. A more detailed analysis of the outer membrane composition is thus warranted.

Previous studies reported that *A. baumannii* often acquires colistin resistance. Here, mutations were found in *lpxA*, *lpxC*, and *lpxD* of the CRAb strains we established. These genes are involved in the early stages of LPS biosynthesis (Raetz and Whitfield, 2002). To the best of our knowledge, only *A. baumannii* and *A. nosocomialis* acquire resistance to colistin through complete LPS deficiency (Moffatt et al., 2010; Vila-Farres et al., 2015). CRAb-6 was created by inserting the IS*Aba11* sequence into *lpxC* (data not shown). This transformation was reported earlier by Moffatt et al. (2011). Thus, the acquisition of colistin resistance by LPS deficiency seems to be a frequently occurring resistance mechanism in *A. baumannii*. A mutation introduced into the two-component *pmrAB* system and LPS modification with phosphoethanolamine both induced colistin resistance in *A. baumannii* (Adams et al., 2009; Beceiro et al., 2011). The *pmrAB* mutation resembled the wild type in terms of growth rate, biofilm formation, and mouse model pathogenicity (Beceiro et al., 2014). Recently, plasmid-mediated colistin resistance gene *mcr* (mobilized colistin resistance) was identified and investigated (Liu et al., 2016). This gene encodes phosphatidylethanolamine transferase, which modifies LPS, and therefore, causes colistin resistance (Hinchliffe et al., 2017). Since neutrophils seemed to exclusively target LPS-deficient *A. baumannii* in the current study, future studies should examine the interactions between neutrophils and LPS modification and colistin resistance in *A. baumannii*.

The LPS-deficient strains are reported to have less fitness (Beceiro et al., 2014; Mu et al., 2016), and so had our CRAb strains (Figures 1, 2A). The LPS-deficient strains might have problems growing on agar. The LPS-deficient strain had fewer colonies on agar even when the OD_600_, indicating the number of planktonic cells in broth, was almost the same value as the parent strain. By establishment of the LPS complement strain of CRAb, we could show that LPS was involved in bacterial fitness (Figure 2A). Furthermore, sensitivities to β-lactam drugs and other antimicrobials were increased in LPS-deficient *A. baumannii* strains compared with those of LPS-positive strains possibly because of the differences in membrane composition of the strains. Our findings suggested that β-lactam drugs may be more effective in patients with neutropenia comorbid with CRAb infection. Therefore, co-use of the colistin and β-lactams may be useful for treatment to *A. baumannii* infection in patients with neutropenia. Moreover, LPS-deficient *A. baumannii* are comparatively more susceptible to disinfectants such as chlorhexidine, sodium dodecyl sulfate, and ethanol than LPS-positive strains (Carretero-Ledesma et al., 2018). A few studies have provided direct evidence that LPS-deficient *A. baumannii* demonstrate lower proliferation, mobility, and biofilm formation capability than their LPS-positive counterparts (Moffatt et al., 2010; Beceiro et al., 2014; Dafopoulou et al., 2015; Carretero-Ledesma et al., 2018; Farshadzadeh et al., 2018).

**FIGURE 7:**
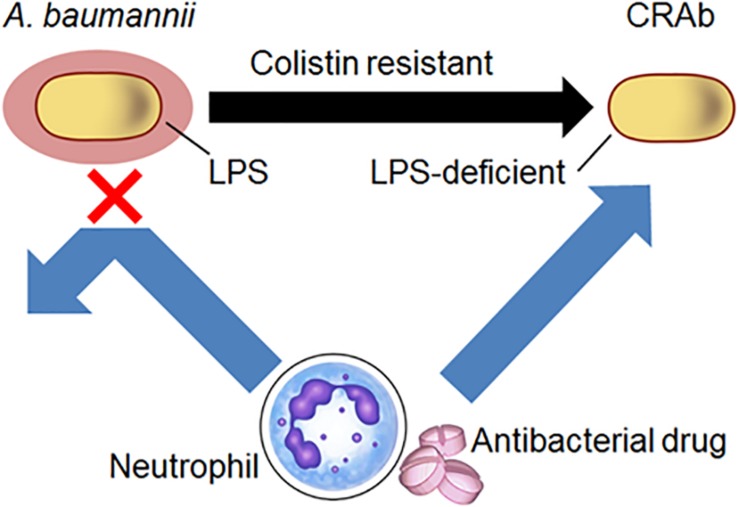
Schematic diagram of the study. *Acinetobacter baumannii* acquire resistance to colistin by LPS deficiency. Neutrophil-derived lysozyme and antibacterial drugs such as β-lactam were killed by LPS-deficient *A. baumannii* strains.

Although *A. baumannii* frequently acquires colistin resistance, LPS-deficient strains are nonetheless susceptible to host immunity, antibacterial drugs, and disinfectants. Clearly, our studies indicate that *A. baumannii* lacking LPS are resistant to colistin. We demonstrated a predilection for neutrophil-derived lysozyme in killing LPS-deficient *A. baumannii* strains and showed that these strains have increased susceptibility to antibacterial proteins from neutrophils and antibacterial drugs such as β-lactam (Figure 7). These findings are substantial in the current combat against colistin-resistant bacteria and control of *A. baumannii* infections in hospitals especially in immunocompromised patients with neutropenia.

## Data Availability Statement

All datasets generated for this study are included in the article/Supplementary Material.

## Ethics Statement

The studies involving human participants were reviewed and approved by the Ethical Review Committee of the School of Medicine of Teikyo University, Tokyo, Japan. The patients/participants provided their written informed consent to participate in this study.

## Author Contributions

GK designed and performed the experiments, analyzed and discussed the data, and wrote the manuscript. TA performed the experiments. NT performed the DNA analysis experiments and discussed the data. YSu, YSa, DK, TH, DY, TK-U, SN, YU, ST-N, TU, and TM-A performed a portion of experiments and discussed the data. MO and YO supervised the study and co-wrote the manuscript.

## Conflict of Interest

The authors declare that the research was conducted in the absence of any commercial or financial relationships that could be construed as a potential conflict of interest.

## Abbreviations

IL: interleukin
LPS: lipopolysaccharide
MDRA: multidrug-resistant *Acinetobacter baumannii*
MOI: multiplicity of infection
PBS: phosphate-buffered saline
PMA: phorbol-12-myristate-13-acetate
ROS: reactive oxygen species
TNF: tumor necrosis factor.
