# Supplementary material for: Lipopolysaccharide-Deficient Acinetobacter baumannii Due to Colistin Resistance Is Killed by Neutrophil-Produced Lysozyme
Source: Front Microbiol. 2020 Apr 17;11:573. doi: 10.3389/fmicb.2020.00573 (PMC7183746; doi:10.3389/fmicb.2020.00573)
Supplement: Supplementary file 1 [file Data_Sheet_1.docx]

**Lipopolysaccharide-deficient *Acinetobacter baumannii* due to colistin resistance is killed by neutrophil-produced lysozyme**

Go Kamoshida^*^, Takuya Akaji, Norihiko Takemoto, Yusuke Suzuki, Yoshinori Sato,　Daichi Kai, Taishi Hibino, Daiki Yamaguchi, Takane Kikuchi-Ueda, Satoshi Nishida, Yuka Unno, Shigeru Tansho-Nagakawa, Tsuneyuki Ubagai, Tohru Miyoshi-Akiyama, Masataka Oda, and Yasuo Ono

*Correspondence: Go Kamoshida: [kamoshida@mb.kyoto-phu.ac.jp](mailto:kamoshida@mb.kyoto-phu.ac.jp)


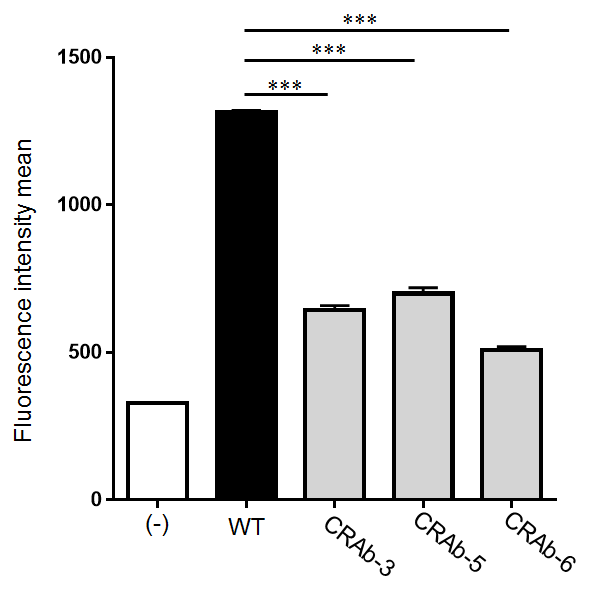


**FIGURE S1 |** Reactive oxygen species (ROS) expression of neutrophils by other colistin-resistance *Acinetobacter baumannii* strains stimulation. ATCC19606 (WT) or colistin-resistant *A. baumannii* strains (CRAb-3, 5, 6) and neutrophils were co-cultured for 1 h and measured ROS by flow cytometry. Data are means ± SD; n=4 per group. ****P* < 0.001.


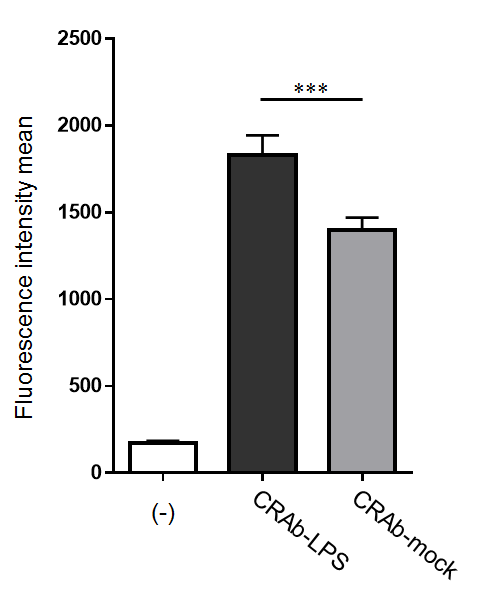


**FIGURE S2 |** Reactive oxygen species (ROS) expression of neutrophils by lipopolysaccharide (LPS) complementation strain of colistin-resistant *Acinetobacter baumannii* (CRAb). CRAb-LPS (LPS complement strain) or CRAb-mock (mock transfectant strain) and neutrophils were co-cultured for 1 h and measured ROS by flow cytometry. Data are means ± SD; n=4 per group. ****P* < 0.001.


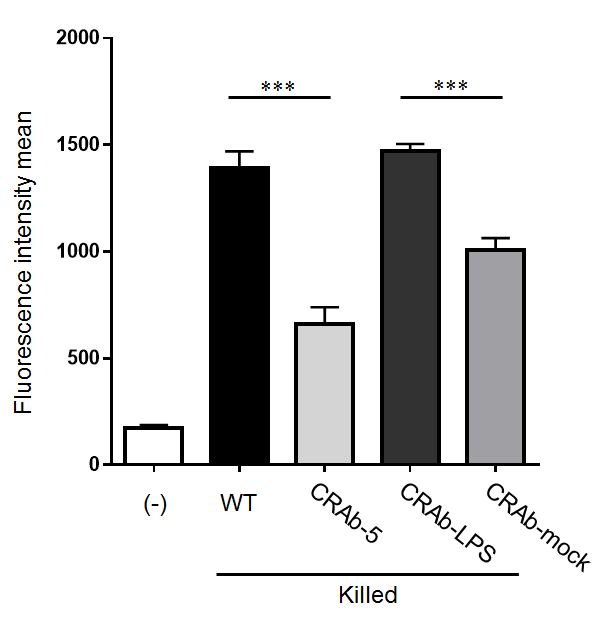


**FIGURE S3 |** Reactive oxygen species (ROS) expression of neutrophils by formalin-killed bacteria. Bacteria were treated with 5% formalin to obtain killed bacteria. ATCC19606 (WT), colistin-resistant *Acinetobacter baumannii* strain (CRAb-5), CRAb-LPS (LPS complement strain), or CRAb-mock (mock transfectant strain) and neutrophils were co-cultured for 1 h and measured ROS by flow cytometry. Data are means ± SD; n=4 per group. ****P* < 0.001.


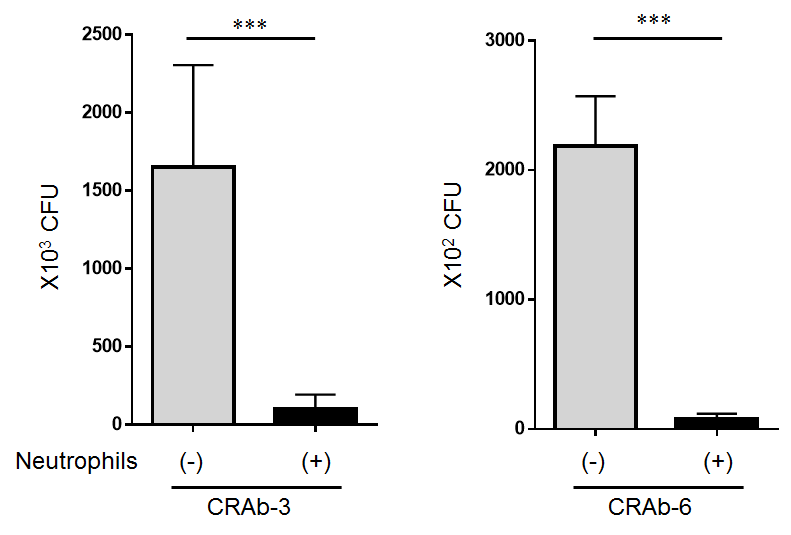


**FIGURE S4 |** Clearance assay of other colistin-resistance *Acinetobacter baumannii* strains by neutrophils. Colistin-resistant *A. baumannii* strains (CRAb-3, 5, 6) and neutrophils were co-cultured for 4 h. Surviving bacteria were then counted. Data are means ± SD; n=6 per group. n.s., not significant, ****P* < 0.001.


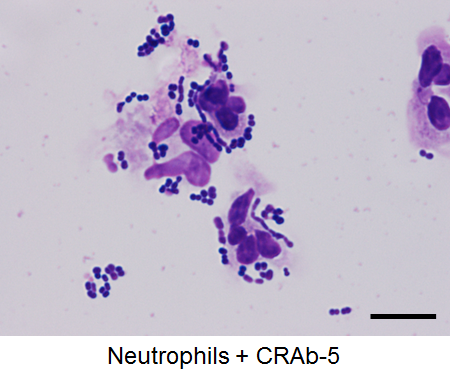


**FIGURE S5 |** Co-cultivating morphology of neutrophils and colistin-resistance *Acinetobacter baumannii* (CRAb). Neutrophils and CRAb-5 were co-cultured (MOI 50) for 1 h. These cells were then fixed and stained with Diff-Quik (according to the manufacturer’s instructions: Sysmex, Kobe, Japan). Scale bar = 10 μm.
